# Supplementary material for: Predicting visual function by interpreting a neuronal wiring diagram
Source: Nature. 2024 Oct 2;634(8032):113–23. doi: 10.1038/s41586-024-07953-5 (PMC11446822; doi:10.1038/s41586-024-07953-5)
Supplement: Supplementary file 6 — CRF and ERF predictions for individual TmY4 and TmY9 cells. Analogous to Supplementary Data 3, but for TmY target types. Shown are the top four monosynaptic pathways, the strongest pathway passing through each of the top ten intermediary types (ranking from Extended Data Fig. 7), and the trisynaptic pathway Tm1–TmY–Dm3–TmY (see the section entitled Prediction of spatial normalization). [file 41586_2024_7953_MOESM6_ESM.zip › DataS4/TmY4/720575940631261132.pdf]

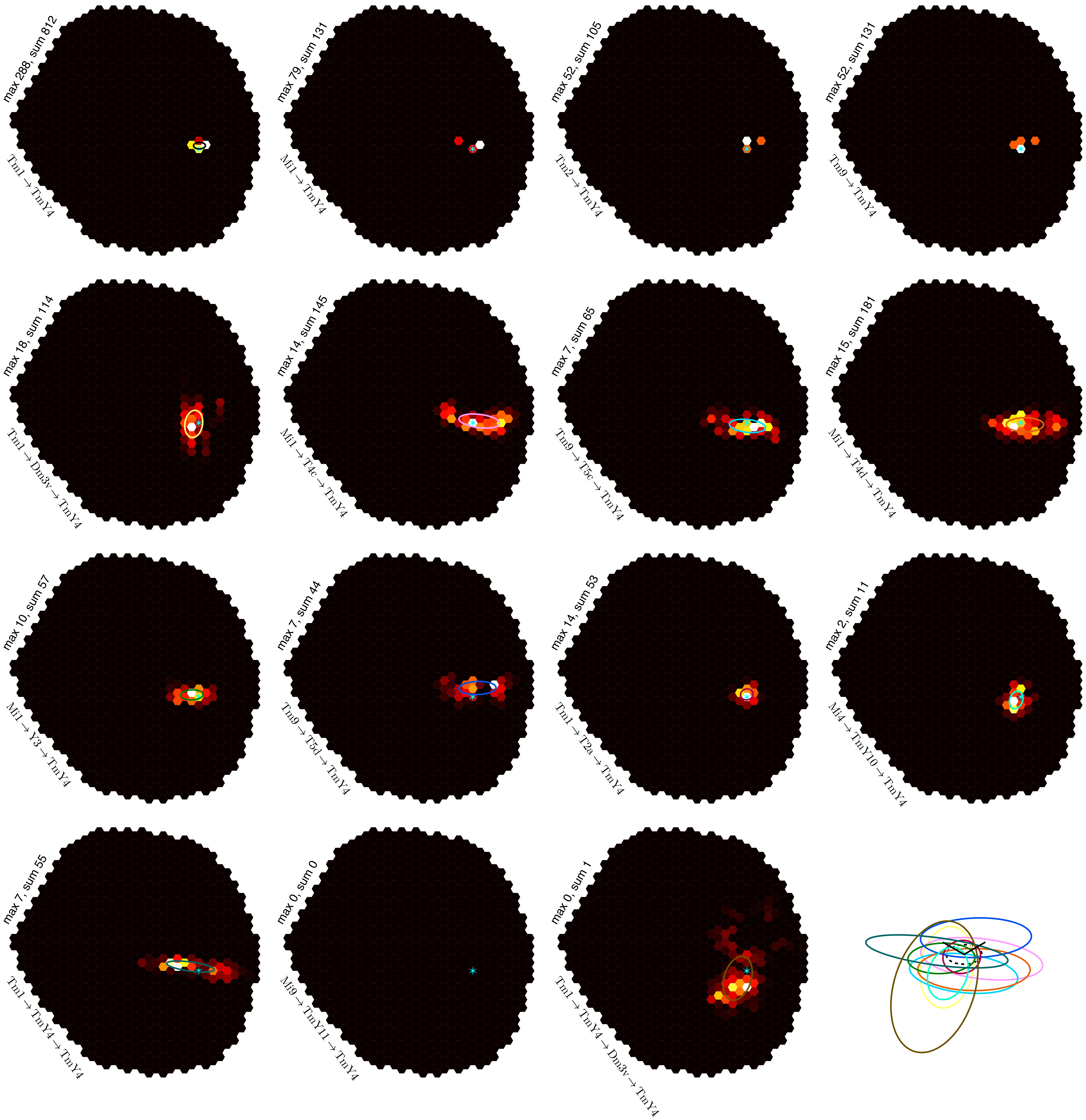
max 288, sum 812
Tm1 → TmY4
max 79, sum 131
Mi1 → TmY4
max 52, sum 105
Tm2 → TmY4
max 52, sum 131
Tm9 → TmY4
max 18, sum 114
Tm1 → Dm3v → TmY4
max 14, sum 145
Mi1 → T4c → TmY4
max 7, sum 65
Tm9 → T5c → TmY4
max 15, sum 181
Mi1 → T4d → TmY4
max 10, sum 57
Mi1 → Y3 → TmY4
max 7, sum 44
Tm9 → T5d → TmY4
max 14, sum 53
Tm1 → T2a → TmY4
max 2, sum 11
Mi4 → TmY10 → TmY4
max 7, sum 55
Tm1 → TmY4 → TmY4
max 0, sum 0
Mi9 → TmY11 → TmY4
max 0, sum 1
Tm1 → TmY4 → Dm3v → TmY4
